# ICU-acquired weakness: what is preventing its rehabilitation in critically ill patients?

**DOI:** 10.1186/1741-7015-10-115

**Published:** 2012-10-03

**Authors:** Christie M Lee, Eddy Fan

**Affiliations:** 1 Interdepartmental Division of Critical Care Medicine, University of Toronto, and the Division of Respirology, Department of Medicine, Mount Sinai Hospital and University Health Network, Toronto, Ontario, Canada; 2 Mount Sinai Hospital, 600 University Avenue, Suite 18-201, Toronto, Ontario, Canada

**Keywords:** critical illness, early ambulation, extracorporeal membrane oxygenation, intensive care units, muscle weakness, physical therapy, rehabilitation

## Abstract

Intensive care unit-acquired weakness (ICUAW) has been recognized as an important and persistent complication in survivors of critical illness. The absence of a consistent nomenclature and diagnostic criteria for ICUAW has made research in this area challenging. Although many risk factors have been identified, the data supporting their direct association have been controversial. Presently, there is a growing body of literature supporting the utility and benefit of early mobility in reducing the morbidity from ICUAW, but few centers have adopted this into their ICU procedures. Ultimately, the implementation of such a strategy would require a shift in the knowledge and culture within the ICU, and may be facilitated by novel technology and patient care strategies. The purpose of this article is to briefly review the diagnosis, risk factors, and management of ICUAW, and to discuss some of the barriers and novel treatments to improve outcomes for our ICU survivors.

## Background

Decreasing mortality following critical illness over the last few decades has shifted the focus from short-term to long-term outcomes in intensive care unit (ICU) survivors. Survivors are often left with profound neuromuscular weakness resulting in persistent functional disability and decreased quality of life for years after hospital discharge [1,2]. Reasons for the development of muscle weakness are multifactorial, including premorbid conditions (for example, malignancy) and ICU-acquired weakness (ICUAW) that may develop as a result of critical illness, systemic inflammation, and certain ICU exposures (for example, enforced bed rest) [3]. This article will focus on reviewing some of the more commonly perceived causes of ICU-acquired weakness, including hyperglycemia, exposure to corticosteroids, and the use of neuromuscular blockers. Currently, no specific treatment exists for ICUAW. Over the last decade, a number of studies have demonstrated the safety and success of early rehabilitation in the ICU [4-6], which may be a promising therapy for the prevention of ICUAW. However, a number of important barriers to advancing our knowledge of the etiology, mechanisms, prevention, and treatment of ICUAW continue to exist. We will discuss some of the issues surrounding early mobility in the ICU, barriers to the implementation of early mobility, and finally, discuss some novel ventilatory strategies to facilitate an awake, calm, and mobile patient in the ICU.

## ICUAW: a rose by any other name

The first reported cases of flaccid paralysis from peripheral neuropathy following sepsis was documented by Bolton *et al*., in 1984 in patients unable to wean from mechanical ventilation [7]. Since then, numerous case series and observational studies of ICUAW have led to an explosion in the number of terms used to describe this syndrome [8]. Concomitantly, there has been an evolving understanding into the various mechanisms by which ICUAW develops, and potential therapeutic targets (for example, physical therapy) for this syndrome, which have been recently reviewed [3,9,10]. However, the absence of a consistent nomenclature has made research in this area challenging, in particular, with two systematic reviews revealing major differences in the definition, diagnosis, reporting of outcomes, and risk factors associated with this syndrome [11,12]. As a result, a new scheme to diagnose and classify ICUAW has been proposed [8]. Broad consensus by clinicians and researchers on a common definition and diagnostic criteria for ICUAW would ensure consistent identification of ICUAW patients and the ability to compare results across studies and patient populations. Ultimately, the diagnosis should begin with routine physical examination of a patient that is weak following an episode of critical illness (Table 1). An initial examination should be performed upon admission to the ICU, with subsequent examinations paired with daily awakenings or when patients show signs of clinical improvement allowing cooperation with a more comprehensive examination. If the patient has an unreliable exam and/or is persistently weak, electrophysiological testing (for example, electromyography (EMG) and nerve conduction studies (NCS)) and/or muscle biopsy may be considered [8].

**Table 1:** Clinical, electrophysiological, and histological features of intensive care unit-acquired weakness (ICUAW)

Investigation	CIP	CIM	CINM
Physical examination	Distal muscle weakness	Proximal muscle weakness	Proximal and distal muscle weakness
	Distal sensory deficit	Normal sensory testing	Distal sensory deficit
	Normal or depressed deep tendon reflexes	Normal or depressed deep tendon reflexes	Depressed deep tendon reflexes
Electrophysiology studies	Decreased CMAP and decreased SNAP	Decreased CMAP and normal SNAP	Decreased CMAP and SNAP
	Normal MUAP	Decreased MUAP	Decreased MUAP
	Normal or near-normal conduction velocity	EMG shows short duration, low amplitude activity	EMG shows short duration, low-amplitude activity
Histology	Axonal degeneration of distal motor and sensory nerves	Thick filament (myosin) loss, type II fiber (fast twitch) atrophy, necrosis	Axonal degeneration and evidence of loss in myosin, type II fiber atrophy, and necrosis

CIM = critical illness myopathy; CINM = critical illness neuromyopathy; CIP = critical illness polyneuropathy; CMAP = compound muscle action potential; EMG = electromyography; MUAP = muscle unit action potential; SNAP= sensory nerve action potential.

## Risk factors and prevention of ICUAW

In the last decade, studies have identified a number of risk factors for ICUAW, but most are small, observational studies with important methodological limitations [9,12]. There are limited and conflicting data regarding the association between severity of illness and ICUAW [9]. Indeed, critical illness neuromyopathy (an important cause of ICUAW) itself is a form of neuromuscular organ failure and similar attention should be placed on prevention and recovery, much the same way that we would approach acute kidney or lung injury.

Hyperglycemia may be an important risk factor for the development of ICUAW. *Post hoc *analyses of two large randomized control trials looking at intensive insulin therapy in both surgical and medical ICUs reported a significant reduction in ICUAW with tight glycemic control [13,14]. However, the results of these secondary analyses and the safety of intensive insulin therapy remains controversial and clinicians should be cautious in using tight glycemic control for the prevention of ICUAW.

There remains substantial controversy regarding the association of ICUAW with two other commonly cited risk factors: corticosteroids and neuromuscular blocking agents (NMBA). Although three prospective observational studies have shown increased risk of ICUAW with corticosteroid exposure [1,15,16], a number of studies have also failed to show a significant association [12,17]. Similarly, despite early evidence to suggest persistent weakness after prolonged infusion of vecuronium [18], subsequent studies evaluating NMBAs have not supported any significant association with ICUAW. Importantly, a recent trial of patients with early acute respiratory distress syndrome (ARDS) randomized to cisatricurium demonstrated a significant reduction in 28-day mortality, without any significant difference in ICUAW as compared to the placebo group [19]. In the end, the decision to use corticosteroids and/or NMBA requires a case-by-case review of the potential benefits and risks, and clinicians must be aware of all the evidence in order to make an informed decision.

## Barriers to early mobility in the ICU

In recent years, a number of studies have documented the safety and feasibility of early mobility in the ICU [4-6]. In addition, early rehabilitation is associated with important reductions in delirium, duration of mechanical ventilation, and improved physical function at hospital discharge [20]. Despite this, only about 25% of all ICU patients receive early therapy [21]. A number of important barriers, both real and perceived, to implementing early rehabilitation at the patient (for example, delirium, hemodynamic instability), provider (for example, staff discomfort, decreased awareness about the importance of early mobility), and institutional (for example, lack of facilities, personnel, equipment) levels have been identified [21,22]. Commonly cited reason for not receiving therapy include oversedation or coma, lack of available rehabilitation staff, and some potentially avoidable including inappropriate vascular access positions, conflict with another planned procedure, and poor sedation management and agitation [21]. While simply pairing physical therapy sessions with sedative interruption may enhance the delivery of early rehabilitation, ultimately, overcoming these barriers will require the creation of an ICU culture that prioritizes early rehabilitation through interdisciplinary coordination, communication, and teamwork. Educational strategies focused on the complications of oversedation (for example, bed rest/immobility, delirium) and its effects on both the short-term and long-term outcomes (for example, ICUAW, neurocognitive/neuropsychiatric morbidity) may help to facilitate culture change. These elements are essential in ensuring the successful and sustained implementation of such a complex intervention. Finally, the barriers, facilitators, and efficacy of early rehabilitation have been evaluated primarily in medical ICU patients; the applicability of these practices in other ICUs (for example, neurologic, trauma, pediatric) require exploration in future clinical trials.

## Early rehabilitation in the ICU: future directions

Novel rehabilitation technology, such as neuromuscular electrical stimulation (NMES) and cycle ergometry, may provide an opportunity for early rehabilitation even when the patient is unable to actively participate (for example, during acute phase of critical illness). In healthy volunteers, NMES can improve or preserve muscle strength by preventing disuse atrophy through stimulated muscle contraction [23], but there are limited data in the critically ill. In a study of mechanically ventilated patients with chronic obstructive pulmonary disease (COPD), those randomized to NMES with physical therapy had improved muscle strength at 28 days and decreased number of days needed to transfer from bed to chair as compared to physical therapy alone [24]. Cycle ergometry, another novel rehabilitation tool can provide passive, active-assisted, or active range of motion exercises [23]. In a recent study of ninety critically ill patients, those randomized to cycle ergometry showed improvements in quadriceps muscle function, 6-minute walk distance, and self-reported physical functioning at hospital discharge [25]. These promising results require confirmation in large, prospective clinical trials.

Enforced bedrest, oversedation, and delirium are common barriers to early rehabilitation in the ICU. Sedation and analgesia are commonly instituted (with or without neuromuscular blockade) in the ICU for management of patient discomfort, anxiety, and asynchrony during mechanical ventilation. Changes in sedation strategies, including novel sedative agents such as dexmedetomidine [26], use of intermittent sedation [27], or no sedation [28] may help to limit oversedation and delirium in the ICU and improve patient wakefulness and availability for physical therapy.

Even more radical might be finding a viable alternative to mechanical ventilation in which sedation and analgesia use can truly be minimized. Extracorporeal membrane oxygenation (ECMO) has become an area of increasing interest following its successful use for H1N1-associated ARDS [29]. By providing extracorporeal gas exchange support, ECMO may mitigate the need for any aggressive mechanical ventilation in patients with respiratory failure. Indeed, a number of groups have exploited this technology to facilitate rehabilitation and ambulation in ICU patients awaiting lung transplantation [30,31]. As this technology continues to improve and miniaturize, it is conceivable that mechanical ventilation could eventually be replaced by ECMO, allowing our critically ill patients to be awake, calm, cooperative, and mobile.

## Conclusions

ICUAW is an important complication that contributes to functional disability and decreased quality of life in ICU survivors. Indeed, it is an important component of the post-intensive care syndrome (PCIS) representing any new or worsening impairment in physical, cognitive, or mental heath status following an acute care hospitalization [32]. The lack of an accepted taxonomy and diagnostic criteria for ICUAW has made it difficult to interpret and compare results across studies. Early rehabilitation may be an important preventative therapy for ICUAW, but there are many important barriers at the patient, provider, and organizational levels which need to be recognized and overcome. Novel rehabilitation (for example, NMES, cycle ergometry) technology may facilitate rehabilitation in patients who cannot actively participate in therapy during the acute phase of their illness. Finally, ECMO may obviate the need for heavy sedation/analgesia and mechanical ventilation, providing a means to provide early rehabilitation to patients with even the most severe forms of respiratory failure in the ICU. The combination of: (1) a coordinated interdisciplinary team; (2) novel advances in both ICU and rehabilitation technology; and (3) a culture that prioritizes early rehabilitation, will help our ICU patients to be awake, calm, cooperative, and mobile, and hopefully translate into substantial improvements in both their short-term and long-term outcomes.

## Competing interests

The authors declare that they have no competing interests.

## Authors' contributions

CML made a substantial contribution to the conception of the mini-review, drafting of the manuscript and revising it critically for important intellectual content. EF conceived the mini-review, and helped in drafting the manuscript and revising it critically for important intellectual content. Both authors read and approved the final manuscript.

## Pre-publication history

The pre-publication history for this paper can be accessed here:

http://www.biomedcentral.com/1741-7015/10/115/prepub
